# Establishing Clonal Cell Lines with Endothelial-Like Potential from CD9^hi^, SSEA-1^−^ Cells in Embryonic Stem Cell-Derived Embryoid Bodies

**DOI:** 10.1371/journal.pone.0000006

**Published:** 2006-12-20

**Authors:** Qizhou Lian, KengSuan Yeo, Jianwen Que, EileenKhiaWay Tan, Fenggang Yu, Yijun Yin, Manuel Salto-Tellez, Reida Menshawe El Oakley, Sai-Kiang Lim

**Affiliations:** 1 Department of Surgery, National University of Singapore Singapore, Singapore; 2 Genome Institute of Singapore Singapore, Singapore; 3 Department of Obstetrics and Gynaecology, National University of Singapore Singapore, Singapore; 4 Department of Pathology, National University of Singapore, Singapore, SingaporeDepartment of Biochemistry, National University of Singapore Singapore, Singapore; Baylor College of Medicine, United States of America

## Abstract

**Background:**

Differentiation of embryonic stem cells (ESCs) into specific cell types with minimal risk of teratoma formation could be efficiently directed by first reducing the differentiation potential of ESCs through the generation of clonal, self-renewing lineage-restricted stem cell lines. Efforts to isolate these stem cells are, however, mired in an impasse where the lack of purified lineage-restricted stem cells has hindered the identification of defining markers for these rare stem cells and, in turn, their isolation.

**Methodology/Principal Findings:**

We describe here a method for the isolation of clonal lineage-restricted cell lines with endothelial potential from ESCs through a combination of empirical and rational evidence-based methods. Using an empirical protocol that we have previously developed to generate embryo-derived RoSH lines with endothelial potential, we first generated E-RoSH lines from mouse ESC-derived embryoid bodies (EBs). Despite originating from different mouse strains, RoSH and E- RoSH lines have similar gene expression profiles (r^2^ = 0.93) while that between E-RoSH and ESCs was 0.83. *In silico* gene expression analysis predicted that like RoSH cells, E-RoSH cells have an increased propensity to differentiate into vasculature. Unlike their parental ESCs, E-RoSH cells did not form teratomas and differentiate efficiently into endothelial-like cells *in vivo* and *in vitro*. Gene expression and FACS analysis revealed that RoSH and E-RoSH cells are CD9^hi^, SSEA-1^−^ while ESCs are CD9^lo^, SSEA-1^+^. Isolation of CD9^hi^, SSEA-1^−^ cells that constituted 1%–10% of EB-derived cultures generated an E-RoSH-like culture with an identical E-RoSH-like gene expression profile (r^2^ = 0.95) and a propensity to differentiate into endothelial-like cells.

**Conclusions:**

By combining empirical and rational evidence-based methods, we identified definitive selectable surface antigens for the isolation and propagation of lineage-restricted stem cells with endothelial-like potential from mouse ESCs.

## Introduction

The pluripotency of mouse embryonic stem cells (ESCs) to differentiate into cells from all three germ layers makes ESCs an ideal source of cells for regenerative therapy for many diseases and tissue injuries [1], [2]. However, this property of ESCs also poses a unique challenge of having to generate therapeutically efficacious quantity of appropriate cell types without being contaminated by potentially deleterious cell types. In this regard, lineage-restricted stem cells as therapeutic agents for regenerative medicine offer several distinct advantages over pluripotent ESCs. One, they are not pluripotent and therefore, cannot form teratoma. Two, lineage-restricted stem cells unlike ESCs, can only differentiate into a limited number of cell types, and differentiation into the target cell types will therefore be more efficient. Third, lineage-restricted stem cells being stem cells will be able to self-renew and therefore could potentially be maintained as a renewable source of cells in a similar manner as ESCs. In addition, lineage-restricted stem cells unlike terminally differentiated cell types can undergo *in situ* differentiation and will therefore integrate better into the recipient target tissue.

ESCs can be induced to differentiate *in vitro* into lineage-restricted stem cells types[3]–[8]. However, to date, only neural stem cells can be clonally expanded in a homogenous culture[9] A major hindrance to the derivation of lineage-restricted stem cells from ESCs is the lack of highly purified tissue stem cells to identify defining surface markers for rare tissue stem cells and this in turn has greatly hinder their isolation. Nevertheless, there have been some success in circumventing this limitation by inserting reporter genes into lineage-specific gene loci [10]–[15], or selecting for surface receptors known to be important in early tissue development e.g. Flk-1 in hematopoiesis and vasculogenesis [16]–[18]. However, CD73+ human mesenchymal stem cell population is, to date, the only lineage-restricted stem cell population to be prospectively isolated from ESC by markers and propagated as a self-renewing population in culture[18]. To isolate clonal ESC-derived lineage-restricted cell lines with endothelial potential and the capacity for self-renewal *in vitro*, we had to use a combination of empirical and rational evidence-based methods to gradually circumvent the lack of definitive selectable markers. We have previously isolated RoSH embryonic cell lines with endothelial potential from early mouse embryos using an empirical approach based on two premises[19]. The first is stochastic and predicts that lineage-restricted stem cells with endothelial potential are likely to be enriched in early embryos as the cardiovascular system is the first functional system in the developing embryos. Second, the self-renewing capability of putative lineage-restricted stem cells will manifest as colonies of steadily dividing and uniformly looking cells. The previously described RoSH cell lines with endothelial potential were derived from 5.5 to 6.5 *dpc* mouse embryos[19]. Since ESC-derived embryoid bodies (EBs) are developmentally analogous to early post-implantation embryos[20], we rationalized that exposing EBs to the empirical protocol for deriving RoSH cell lines could also generate RoSH-like cells. Several RoSH-like lines termed E-RoSH lines were generated; these lines were highly similar to RoSH lines. They do not express pluirpotency-associated genes, failed to induce teratomas in immunodeficient mice and differentiate readily into endothelial-like cells. Using genome-wide gene expression profiling and FACS analysis, we identified RoSH/E-RoSH cells as CD9^hi^, SSEA-1^−^ while ESCs were CD9^lo^, SSEA-1^+^. The selection criteria of CD9^hi^ and SSEA-1^−^ were stringent enough to isolate putative E-RoSH cells from differentiating ESC culture for the establishment of E-RoSHL cell lines.

## Methods

### Derivation of E-RoSH cell lines

E14 ESCs were induced to differentiate to form EBs using the methycellulose-based approach[21]. Day 3 to day 6 EBs were harvested, dissociated into single cell suspensions by collagenase digestion [22] and plated on at a density of 1–5×10^5^cells per 10 cm feeder plate in RoSH media consisting of 400 ml DMEM (Cat No: 10313-021) , 100 ml FCS (Cat No: 1049-024), 5 ml Penicillin-streptomycin-glutamine (Cat No: 10378-016), 5 ml non-essential amino acids (Cat No: 11140-050), 0.5 ml β-mercaptoethanol (Cat No: 21985-023) (Gibco, Grand Island, New York). After about a week, the cells proliferated and differentiated into a complex mixture of cell types. Colonies of rapidly dividing cells resembling embryo-derived RoSH cells were picked and expanded sequentially to a 48-well plate, 24-well plate, 6-well plate and then a 10 cm plate. The culture from each colony was named E-RoSH1, 2, 3…in the sequence in which each culture was established. Each of these cell cultures were then replated at 10–100 cells per 10 cm plate. Colonies were then selected and expanded to establish sublines that were named based on their parental lines e.g. E-RoSH1.1, 1.2, 1.3, etc. For suspension cultures, 1×10^6^ cells were plated on 10 cm bacterial Petri dishes that were placed on an orbital shaker. Alkaline phosphatase assay were performed using assay kits from Chemicon (Temecula, California) and Bioassay Systems (Hayward, California). Chromosomes counting was performed as previously described[22]

### RT-PCR analysis

Total RNA was prepared using standard protocols and were quantified using RiboGreen RNA Quantification kit (Molecular Probes, Eugene, Oregon). Quantitative RT-PCR was performed using TaqMan® primers (Applied Biosystems, Foster City, CA). The Taqman primer ID for each gene analyzed was Pou5f1-Mm00658129_gH; Sox2-Mm00488369_s1; Tdgf1-Mm00783944_g1; Hesx1-Mm00439312_g1; Gata4-Mm00484689_m1; Kit-Mm00445212_m1; Pdgfra-Mm00440701_m1; Tek-Mm00443242_m1; Afp-Mm00431715_m1; Fabp2-Mm00433188_m1; Foxa2-Mm00839704_mH; Sox17-Mm00488363_m1; Isl1-Mm00627860_m1; Neurog3-Mm00437606_s1; Pax6-Mm00443072_m1; Pcsk1Mm00479023_m1.

### Western Blotting

Total protein was isolated using standard protocols and 30 µg cell lysates were separated by SDS-PAGE, transferred to nitrocellulose membrane and incubated with goat anti-Oct3/4 (Santa Cruz Biotechnology,sc-8628), goat anti-Sox2(sc-17320), rabbit anti-Nanog ( Chemicon, AB5731), or mouse anti-β-actin antibodies (Chemicon, MABP501) followed by incubation of the relevant HRP-conjugated secondary antibodies and then a HRP enhanced chemiluminescent substrate, ECS (Pierce, Rockford, IL) for visualization.

### Illumina gene chip analysis

Total RNA (2 µg) from each sample of E-RoSH2.1 p6 and p10, E-RoSH3.2 p2 and p7, RoSH2 p9 and p13, E-RoSHL1 p5 and p6, and E14 mESCs p15 and p16 cultures were converted to biotinylated cRNA using the Illumina RNA Amplification Kit (Ambion, Inc., Austin, TX) according to the manufacturer's instructions. Samples were purified using the RNeasy kit (Qiagen, Valencia, CA). Hybridization to the Sentrix Mouse Refseq-8 Expression BeadChip (Illumina, Inc., San Diego, CA), washing and scanning were performed according to the Illumina BeadStation 500x manual. The data were extracted, normalized and analyzed using Illumina BeadStudio provided by the manufacturer. Transcript signals that were below the limit of detection (LOD) at 99% confidence were eliminated as genes not expressed.

### *In vitro* endothelial differentiation

Endothelial differentiation of E-RoSH cells and acetylated LDL uptake by differentiated E-RoSH cells were performed as previously described [19]. In vitro differentiated E-RoSH vascular structures were fixed in formalin, and immunostained for vWF using polyclonal, rabbit-generated antibody and Envision+ System-peroxidase (DakoCytomation, Gostrup, Denmark) and counterstained with Mayer's hematoxylin, or Tie-2 and CD34 using rabbit anti-Tie-2 and goat anti-CD34 (Pharmingen, San Diego, California) respectively, followed by FITC-conjugated anti-rabbit/goat antibody (Chemicon, Temecula, California), and counterstained with PI.

### *In vivo* endothelial differentiation

1×10^6^ ESCs were transplanted subcutaneously into SCID mice. At three weeks when ESC-derived tumors were about 0.5–1 cm in diameter, 1×10^5^ E-RoSH cells labeled with Qdot® nanocrystals (655 nm emission) using a Qtracker® Cell Labeling Kit (Quantum Dot Corp, Hayward, CA) were injected into the ESC-derived teratoma. Three days later, the mice were euthanized with an overdose of anesthesia and the tumors were removed. The tumors were fixed in 4% paraformaldehyde and cryosectioned at 20 µm thickness. The sections were assayed for Tie-2 immunoreactivity using using rabbit anti-Tie-2 (Santa Cruz Biotechnology, Cat No:sc-9026) followed by FITC-conjugated rabbit anti-rat antibody (Chemicon, Temecula, California), and counterstained with DAPI. The sections were analyzed by confocal microscopy.

### Surface antigen analysis during differentiation

Cell surface antigens on E-RoSH cells before and 60 hours after induction of differentiation on matrigel were analyzed using flow cytometry. The cells were tryspinized for 5 minutes, centrifuged, resuspended in culture media and incubated in a bacterial culture dish for 2–3 hours in a 37°C, 5% CO_2_ incubator. The cells were then trypsinized for 1 minute, centrifuged, washed with PBS, fixed in 4% paraformaldehyde for 0.5 hour at room temperature, washed and blocked in 2%FCS for 0.5 hour at room temperature with agitation. 1.5×10^5^cells were then incubated with each of the following conjugated monoclonal antibodies: Tie-2, Flk-1, CD34, c-Kit, Thy-1, Sca-1 (PharMingen, San Diego, CA) for 90 mins at room temperature. After incubation, cells were washed and resuspended in PBS. Nonspecific fluorescence was determined by incubation of similar cell aliquots with isotype-matched mouse monoclonal antibodies (PharMingen, San Diego, CA). Data were analyzed by collecting 20,000 events on a Cyan LX with WinMDI software. (Dako North America, Inc., Carpinteria, CA).

### Sorting for putative E-RoSH cells

Sorting for CD9^hi^ SSEA-1^−^ was performed one week after 5 days old EBs have been plated on gelatinized plates. The cultures were trypsinized for 7 mins, neutralized, centrifuged, resuspended in the culture media and then plated on bacterial culture dish. After 2 hours at 37°C in CO_2_ incubator, the cells were harvested, washed with PBS and incubated with CD9-PE and SSEA-1-FITC (PharMingen, San Diego, California) for 40 mins at room temperature. The cells were then washed with PBS and sorted on a FACS Aria using FACS Diva software (BD Biosciences Pharmingen, San Diego, CA).

## Results

### Derivation of RoSH-like lines from ESCs

**Figure 1:**
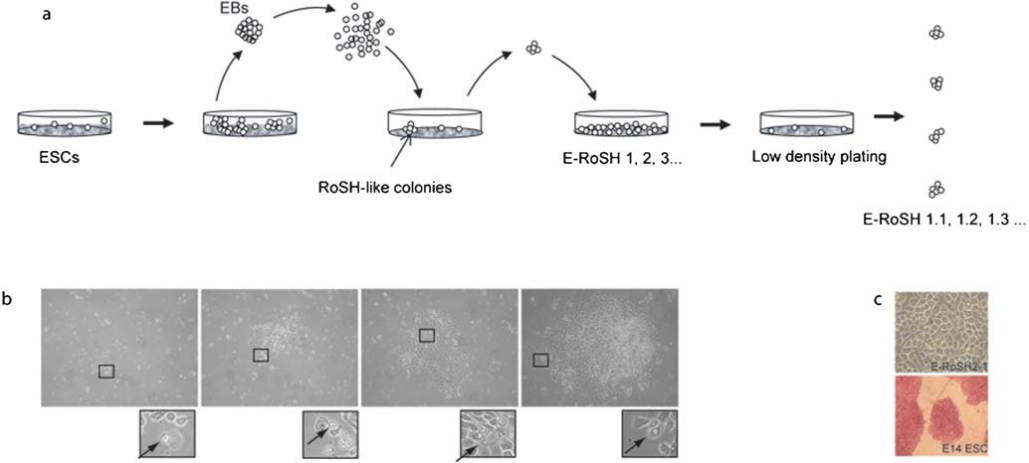
Derivation of E-RoSH cell lines. (a) ESCs were plated singly on methycellulose based media to form EBs. At day 3–6, EBs were harvested, dissociated by collagenase and cultured as a monolayer on gelatinized feeder plate. RoSH-like colonies with adherent fibroblast-like cells and ring-like structures were selected and propagated on gelatinized plates to generate E-RoSH 1, 2, 3… Each of the cultures were then plated at a low density of 10–100 cells per 10 cm plate and single RoSH like colonies were picked to established sublines, E-RoSH 2.1, 2.2, 2.3. .. etc; b) A putative RoSH-like colony consisting of adherent short fibroblast-like cells with characteristic ring-like cells (inset) expanding over time; c) Alkaline phosphatase staining of E-RoSH2.1 and its parental E14 ES cells.

**Figure 2:**
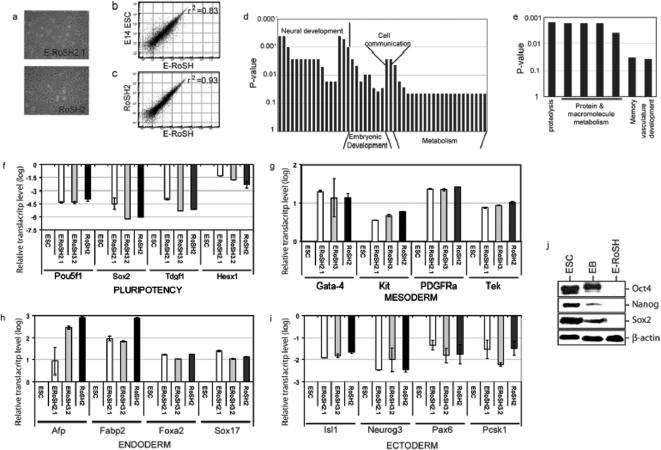
Characterisation of E-RoSH cell lines. a) Cellular morphology of E-RoSH2.1 and RoSH2 cells in sub-confluent cultures; b,c) Pairwise comparison of global gene expression between E14 ESCs and E-RoSH2.1/E-RoSH3.2, and RoSH2 and E-RoSH2.1/E-RoSH3.2. Global gene expression analysis of E14 ESCs, E-RoSH, RoSH were performed by hybridizing total RNA from two biological samples each of E14 ESCs, E-RoSH2.1, E-RoSH3.2, and RoSH2 with Illumina BeadArray containing about 24,000 unique features; d) Biological processes in which 1115 highly expressed genes in ESCs have statistically significant higher frequencies (p<0.05). The biological processes were (*left to right*): neuron differentiation, neuron development, nervous system development, neuron maturation, nerve ensheathment, cellular nerve ensheathment, ionic insulation of neurons by glial cells, myelination, transmission of nerve impulse regulation of action potential, neurophysiological process, neuron morphogenesis during differentiation, neurite morphogenesis, axonogenesis, cell development, development, system development, cell maturation, cell differentiation, cellular morphogenesis, regulation of gene expression, epigenetic Imprinting, gametogenesis, morphogenesis, sexual reproduction, cell-cell signalling, cell communication, metabolism, cell organization and biogenesis, regulation of biological process, macromolecule catabolism, carbohydrate catabolism, cellular carbohydrate catabolism, monosaccharide catabolism, hexose catabolism, glucose catabolism, glycolysis, cellular macromolecule catabolism, cellular catabolism, alcohol catabolism, carbohydrate metabolism, cellular carbohydrate metabolism, monosaccharide metabolism, hexose metabolism, glucose metabolism, main pathways of carbohydrate metabolism alcohol metabolism, generation of precursor metabolites and energy, energy derivation by oxidation of organic compounds; e) Biological processes in which 1263 highly expressed genes in ESCs have statistically significant higher frequencies (p<0.05). The biological processes were (*left to right*): proteolysis, protein metabolism, cellular protein metabolism, cellular macromolecule metabolism, macromolecule metabolism, memory and vasculature development; f,g, h, i) Relative gene expression analysis by quantitative RT-PCR analysis. The expression level was normalized against that of ESCs and expressed as a logarithmic function; j) Western blot analysis for pluripotency-associated gene products in cell extracts of ESCs, EBs and E-RoSH2.1 cells.

To derive RoSH-like cells from ESCs, we rationalized that since our derivation of RoSH cell lines was most optimal when using 5.5 to 6.5 *dpc* mouse embryos[19], 3–6 days old ESC-derived embryoid bodies (EBs) that are developmentally analogous to early post-implantation embryos for the derivation of RoSH-like cells would also be optimal for the derivation of RoSH-like cells from ESCs[20]. Briefly, 3–5 days old ESC-derived EBs were dispersed into single cells by collagense digestion and passaged several times on gelatinized culture dish. After one or two weeks, colonies of RoSH-like cells were often discernible enough to be picked and clonally expanded (fig 1a; Methods). We observed that 5 days old EB was most efficient in generating RoSH-like colonies (fig 1b). Nine ESC-derived RoSH (E-RoSH) lines were independently derived, five from CSL3 ES cell line and four from E14 ES cell line. Unlike its parental ESCs, E-RoSH cells did not have detectable alkaline phosphatase activity (fig 1c). Single cell plating of two lines E-RoSH2 and E-RoSH3 generated clonal sub lines E-RoSH2.1 and E-RoSH3.2 respectively and these sublines were used for further characterisation (fig 1a). Both E-RoSH2.1 and E-RoSH3.2 lines have a strong modal chromosome number of 40 at passage 10 after subcloning. E-RoSH cells were morphologically similar to RoSH cells (fig 2a). Despite being of different mouse genetic background, gene expression profiles of E-RoSH2.1 and E-RoSH3.2 cells of 129Sv mouse strain and RoSH2 cells of C57BL6/J mouse strains were highly correlated (r^2^ = 0.93) (fig 2b). As a point of reference, technical replicates using the same RNA samples performed at 2–3 months apart have a correlation coefficient of 0.96–0.98. The gene expression profiles of E-RoSH2.1 and E-RoSH3.2 cells and their parental E14 ESCs have a lower correlation coefficient, r^2^ = 0.83 (fig 2c). 1115 genes were found to be expressed in ESCs at >2.5 fold higher than in E-RoSH cells. Statistically significant higher frequencies of these genes (p<0.05) were associated with biological processes that can be broadly classified as neural differentiation, embryo development and metabolism (fig 2d), suggesting that the differentiation potential especially neural differentiation potential of E-RoSH cells was reduced relative to ESCs. In contrast, among the 1263 genes that were expressed in E-RoSH cells at >2.5 fold higher than in ESCs, a statistically significant higher frequency of genes (p<0.05) was associated with biological processes involved in proteolysis, protein metabolism, cellular protein metabolism, cellular macromolecule metabolism, macromolecule metabolism, memory and vasculature development (fig 2e). Together, these global gene expression analyses suggest that unlike their parental pluripotent ESCs, E-RoSH cells have a reduced neural differentiation potential and may have lost their pluripotency. Their gene expression profile also suggested that they are better poised to form vasculature than their parental ESCs.

Real time RT-PCR analysis of representative genes confirmed that expression of pluripotency associated genes as Pou5f1, Hesx1, Sox-2 and Tdgf1 were at least 10^2–7^ lower in E-RoSH2.1 and E-RoSH3.2 than its parental E14 ESC (fig. 2f)[23], [24]. In contrast, expression of several genes that are important in mesodermal differentiation e.g. Gata-4, Kit, Tek, Pdgfra (fig. 2g), and endodermal differentiation e.g. Afp, Fabp2, Foxa2 and Sox17 were elevated (fig. 2h). Consistent with the global gene expression analysis, many genes known to be induced in early ectodermal differentiation of ESCs such as Isl1, Neurog3, Pax6 and Pcsk1 were not upregulated (fig. 2i). The much reduced expression of pluripotency-associated genes in E-RoSH cells e.g. Pou5f1, Sox-2 and Nanog was confirmed by the undetectable level of their protein products in total cellular extract from E-RoSH cells (fig. 2j).

### Differentiation of E-RoSH lines

**Figure 3:**
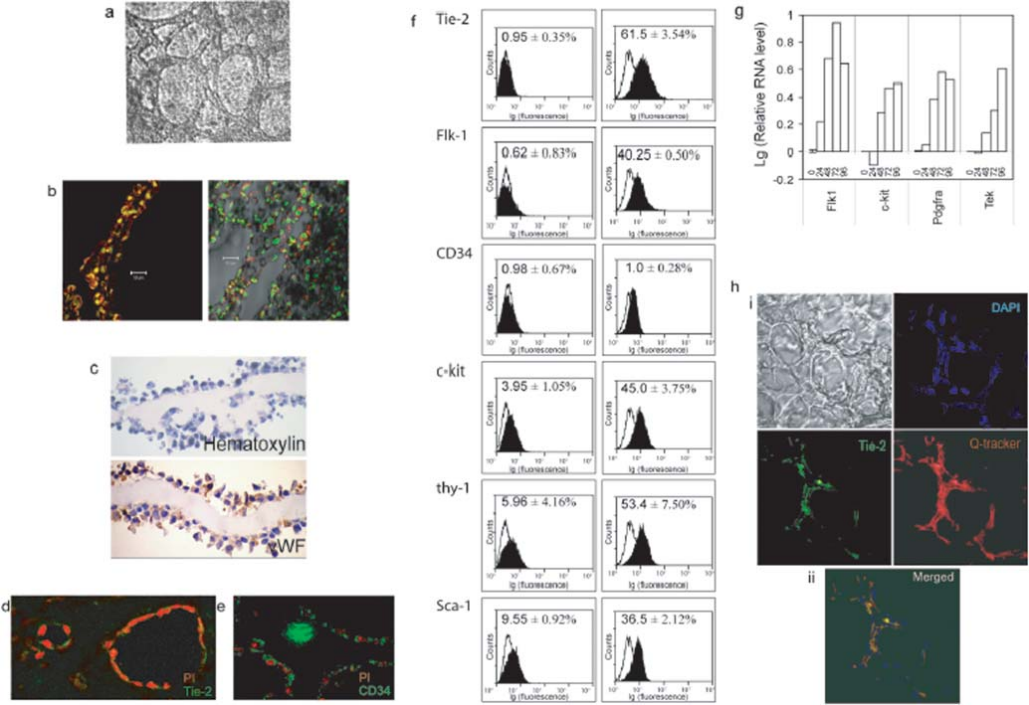
Differentiation of E-RoSH cells. *In vitro* differentiation a) Morphology of E-RoSH2.1 cell culture two weeks after plating E-RoSH2.1 cells on matrigel coated plate, b) The patent E-RoSH2.1 derived tubular structures were labeled with CFDA, a cytoplasmic green fluorescent dye (Molecular Probe, Eugene, OR) and propidium iodide, and viewed by confocal microscopy (left panel). The tubular structures were incubated with acetylated red fluoresecent diI-labelled LDL (Molecular Probe, Eugene, OR) for 24 hours and counterstained with SYTOX Green™, a green fluorescent nuclear dye (Molecular Probe, Eugene, OR) before analysis by confocal microscopy (right panel). c–e) Immunoreactivity for vWF, Tie-2 and CD34 on sections of E-RoSH2.1 derived tubular structures. vWF immunoreactivity was visualized using HRP-based detection system. Brown precipitates indicate positive staining. The nuclei were stained with Mayer's hematoxylin. CD34 and Tie-2 immunoreactivities were detected using secondary antibodies conjugated with FITC. Nuclei were counterstained with PI. f) Flow cytometry analysis of E-RoSH2.1 cells for endothelial markers before *(right panels)* and 60 hours after *(left panels)* induction of differentiation by plating cells on matrigel. Nonspecific fluorescence was determined by incubation of similar cell aliquots with isotype-matched mouse monoclonal antibodies or with secondary antibody alone. g) Gene expression during *in vitro* differentiation of E-RoSH2.1 cells on matrigel as measured by quantitative RT-PCR analysis. Relative gene expression is normalized against that at time 0 and expressed as a logarithmic function. h) *In vivo* differentiation. 1×10^5^ E-RoSH2.1 cells labeled with Qdot® nanocrystals (655 nm emission) were injected into a ESC-derived teratoma that was induced in SCID mice. Three days later, the mice were euthanized and the tumors were removed. The tumors were fixed in 4% paraformaldehyde and cryosectioned at 20 µm thickness. The sections were assayed for Tie-2 immunoreactivity using rabbit anti-Tie-2 followed by FITC-conjugated goat anti-rabbit antibody, and counterstained with DAPI. i. A typical section of a capillary plexus in the teratoma as viewed by phase contrast microscopy (*top left panel*), stained with DAPI, a nuclear stain (*top right panel*), stained with using rabbit anti-Tie-2 (*bottom left panel*), and cells labelled with Qdot (*bottom right panel*). ii. Merging of the three fluorescent stains. Yellow fluorescence indicates co-localisation of Qdot and FITC-conjugated antibody.

To evaluate the *in silico* gene expression prediction that E-RoSH cells are poised to differentiate into vasculature, E-RoSH cells were plated directly onto matrigel. Unlike their parental ESCs which can be propagated and maintained in an undifferentiated state when plated on matrigel in ESC media [25], plating of E-RoSH2.1 and 3.2 cells or RoSH2 cells on matrigel in RoSH cell media was sufficient to induce endothelial differentiation and the formation of vascular-like tubules that covered the entire tissue culture dish[19]. These E-RoSH2.1-derived tubules were patent and cells lining the lumen endocytosed acetylated LDL (fig 3b) and were immunoreactive for vWF (fig. 3c), Tie-2 (fig. 3d), and CD34 (fig. 3e)[26] (data not shown for E-RoSH3.2). Within 60 hours after plating on matrigel, the proportion of E-RoSH2.1 cells that expressed Tie-2 and Flk-1, the key receptors in the regulation of embryonic vascular development[27] was dramatically increased from <1% of undifferentiated E-RoSH2.1 cells to 61.5% and 40.25%, respectively (fig. 3f). The proportion of cells that expressed other endothelial surface antigens such as c-kit, thy-1, Sca-1 were also increased. There was minimal increase in CD34+ cells within the first 60 hours after induction of differentiation. Expression of endothelial genes such as Flk-1, Kit, Pdgfra and Tek was also increased with increased immunoreactivity against the gene products (fig. 3g).

To confirm the loss of pluripotency in E-RoSH cells, 1×10^6^ E-RoSH2.1 or 3.2 cells were subcutaneously transplanted into SCID or immunodeficient Rag1 −/− mice. Unlike similar transplantation of the parental ESCs that invariably generated a 2 cm teratoma within three weeks, E-RoSH cells did not induced any tumor formation during a two to nine-months' observation. To induce endothelial differentiation of E-RoSH cells *in vivo*, the E-RoSH 2.1 cells were first labelled with a long term, cell-permeable fluorescent cell label, Q-tracker, and then transplanted into a growing parental ESC-derived teratoma. The rationale was that a teratoma where ESCs are actively differentiating into tissues of all three germ layers is likely to provide a microenvironment conducive for differentiation into tissues of the three germ layers including vascular tissues. Three days after transplantation, E-RoSH cells were found to be extensively incorporated into the capillary plexuses of the teratomas and were immunoreactive for Tie-2 (fig. 3h). E-RoSH cells were also dispersed throughout the teratomas but we could not identify the tissue type that they were associated with.

### Identification of CD9 as a selectable marker for the isolation of E-RoSH cell lines

**Figure 4:**
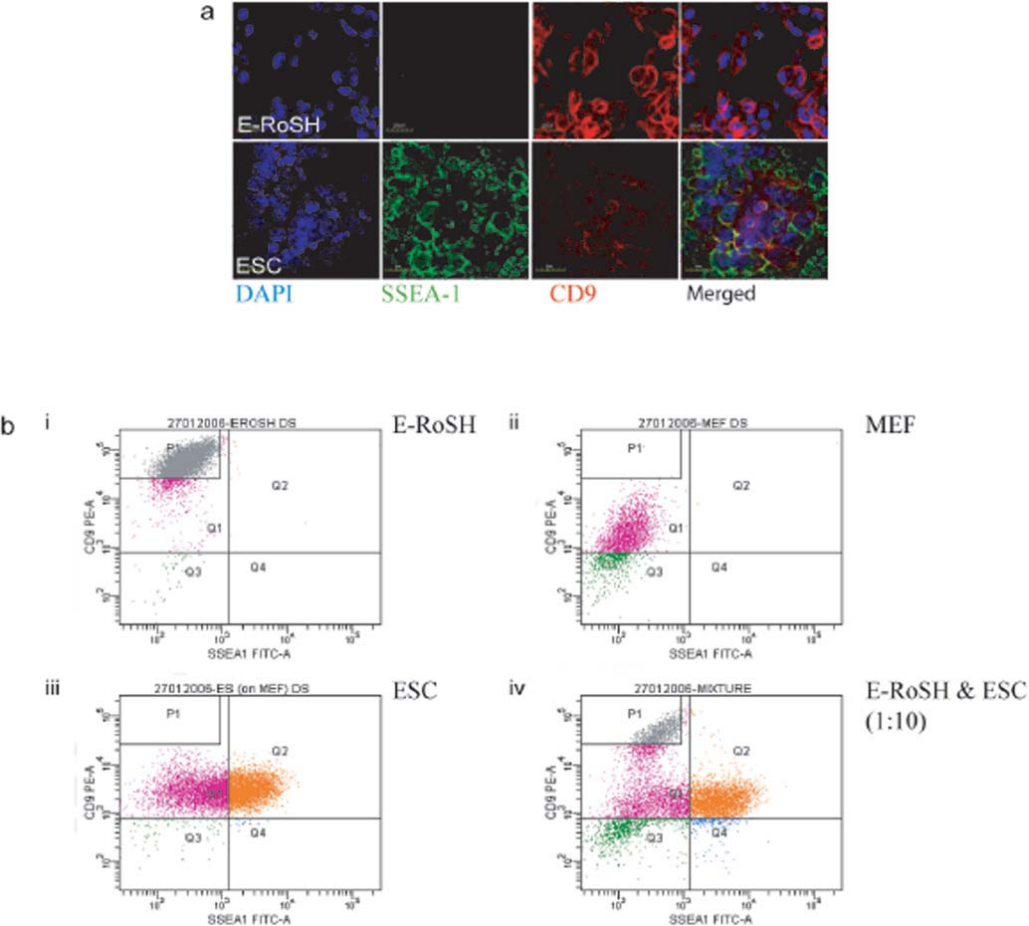
Identifying selectable surface antigens for the isolation of putative RoSH-like cells from differentiating ESCs. a) Confocal microscopy of E-RoSH2.1 cells *(top)* and E14 ESCs *(bottom)*. The cells were counterstained with DAPI, a nuclear stain after immunostaining for anti-SSE4-1 antibody conjugated with FITC and anti-CD9 antibody conjugated with PE. b) FACS analysis of i) E-RoSH2.1 cells, ii) E14 ESCs, iii) murine embryonic fibroblast (MEF) and iv) 10∶1 mixture of E14 ESCs and E-RoSH2.1 cells. The cells were labelled with anti-CD9 antibody conjugated with PE and anti-SSE4-1 antibody conjugated with FITC, and analyzed on a FACS Aria using FACS Diva software (BD Biosciences Pharmingen, San Diego, CA).

**Figure 5:**
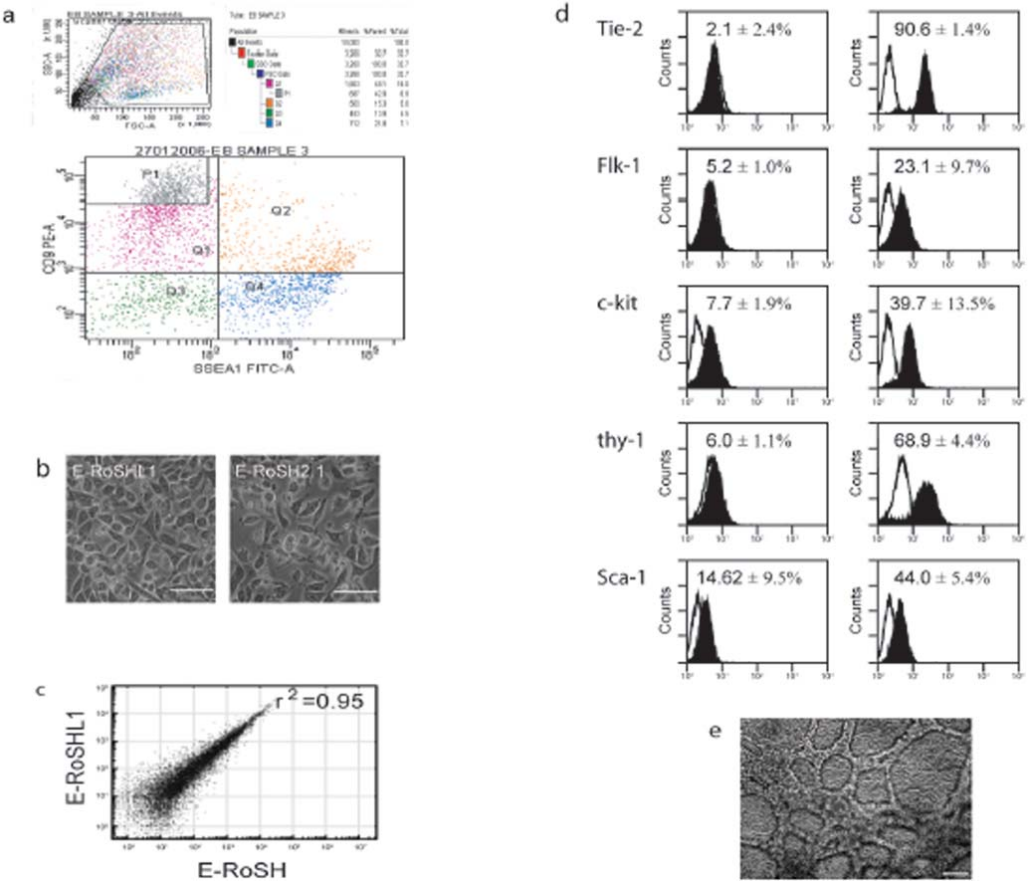
Derivation of E-RoSHL1 cell line from CD9^hi^, SSEA1^lo^ cell population in EB culture. a) One week after plating 5 day old EBs on gelatinized culture plates, the cells were harvested, labelled with anti-CD9 antibody conjugated with PE and anti-SSE4-1 antibody conjugated with FITC, and sorted. CD9^hi^, SSEA1^lo^ cells in the P1 quadrant were selected as putative RoSH cells and plated on gelationized feeder plate; b) Morphology of semi-confluent E-RoSHL1 and E-RoSH2.1. Bar represent 15 µm; c) Pairwise comparison of global gene expression between E-RoSHL1 and E-RoSH cells. Global gene expression analysis were performed by hybridizing total RNA from two biological samples each of E-RoSHL1, E-RoSH2.1 and E-RoSH3.2 with Illumina BeadArray containing about 24,000 unique features; d) Flow cytometry analysis of E-RoSHL1 cells for endothelial markers before *(righ panels)* and 60 hours after *(left panels)* induction of differentiation by plating cells on matrigel. Nonspecific fluorescence was determined by incubation of similar cell aliquots with isotype-matched mouse monoclonal antibodies or with secondary antibody alone; e) Morphology of E-RoSHL1 culture one week after induction of differentiation by plating on matrigel. Bar represents 50 µm.

To develop a more definitive and reproducible protocol for the isolation of E-RoSH cell lines, we analyzed the gene expression profiles of RoSH2, E-RoSH2.1, E-RoSH3.2 and E14 ESCs by Illumina microbead array (Data S1) for surface antigens that were differentially expressed in RoSH-like cells and ESCs, and that already have commercially available antibodies. We therefore generated a list of top 20 highly expressed genes encoding CD antigens (Table 1). Despite the relatively high transcript levels of PDGFRa, Tie2, c-kit, CD59, CD63, CD44 and CD24 in RoSH and E-RoSH cells, they were not immunoreactive against PDGFRa, Tie2, c-kit, CD59, CD63, CD44 and CD24 (data not shown). Gene expression analysis also predicted that CD9, a plasma membrane-bound tetraspanin in mouse ESCs[28], was more highly expressed in RoSH and E-RoSH cells and this was confirmed by confocal muicroscopy of CD9 immunostained E-RoSH2.1 and E14 ESCs (fig. 4a). Flow cytometry determined that CD9 immunoreactivity in E-RoSH2.1 or RoSH2 cells was 10^1–2^ fold higher than that in E14 ESCs (fig. 4b) (data not shown for RoSH2 cells). In addition, expression of SSEA-1, an ESC-specific antigen[29], [30] was absent on E-RoSH2.1 cells (fig. 4a, b). Based on the expression pattern of CD9 and SSEA-1, flow cytometry readily resolved a 1:10 mixture of E-RoSH2.1 cells and E14 ESCs into two distinct populations, CD9^hi^, SSEA1^−^ and CD9^lo^, SSEA1^+^ (fig. 4b). To test if putative RoSH cells in adherent monolayer culture generated by plating 5 day EBs could be isolated using CD9 and SSEA-1 as selectable markers, the heterogenous differentiating EB culture was sorted for CD9^hi^, SSEA1^−^ cells that routinely constituted about 1–10% of these heterogenous cultures (fig. 5a). One isolated CD9^hi^, SSEA1^−^ cell population was plated on a gelatnized plate. It generated a morphologically homogenous RoSH-like cell culture (fig. 5b) termed E-RoSHL1. E-RoSHL1 has a population doubling time of about 12–15 hours, and a gene expression profile that was almost identical to that of E-RoSH with r^2^ = 0.95 (fig. 5c). Like E-RoSH cells, plating of E-RoSHL1 cells on matrigel dramatically increased expression of endothelial markers such as Tie-2, Flk-1, c-kit, Sca1 and thy1 within 60 hours (fig. 5d) and they formed a network of vascular structures that covered the entire culture dish after one week (fig. 5e).

**Table 1:**
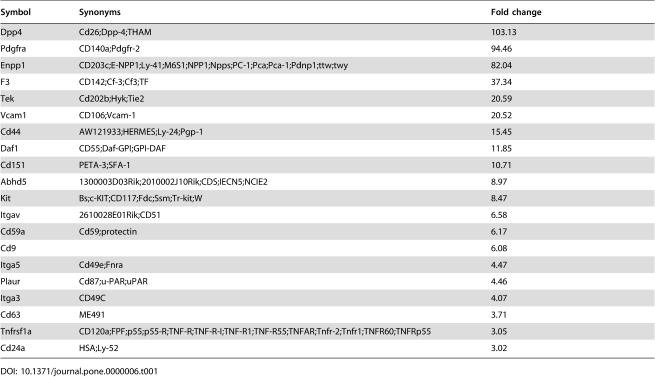
List of top 20 highly expressed genes encoding CD antigens generated by comparing gene expression profiles of E-RoSH and RoSH cells with E14 ESCs.

Symbol	Synonyms	Fold change
Dpp4	Cd26;Dpp-4;THAM	103.13
Pdgfra	CD140a;Pdgfr-2	94.46
Enpp1	CD203c;E-NPP1;Ly-41;M6S1;NPP1;Npps;PC-1;Pca;Pca-1;Pdnp1;ttw;twy	82.04
F3	CD142;Cf-3;Cf3;TF	37.34
Tek	Cd202b;Hyk;Tie2	20.59
Vcam1	CD106;Vcam-1	20.52
Cd44	AW121933;HERMES;Ly-24;Pgp-1	15.45
Daf1	CD55;Daf-GPI;GPI-DAF	11.85
Cd151	PETA-3;SFA-1	10.71
Abhd5	1300003D03Rik;2010002J10Rik;CDS;IECN5;NCIE2	8.97
Kit	Bs;c-KIT;CD117;Fdc;Ssm;Tr-kit;W	8.47
Itgav	2610028E01Rik;CD51	6.58
Cd59a	Cd59;protectin	6.17
Cd9		6.08
Itga5	Cd49e;Fnra	4.47
Plaur	Cd87;u-PAR;uPAR	4.46
Itga3	CD49C	4.07
Cd63	ME491	3.71
Tnfrsf1a	CD120a;FPF;p55;p55-R;TNF-R;TNF-R-I;TNF-R1;TNF-R55;TNFAR;Tnfr-2;Tnfr1;TNFR60;TNFRp55	3.05
Cd24a	HSA;Ly-52	3.02

## Discussion

In this report, we described a strategy for generating non-pluripotent cell lines with endothelial differentiation potential by using a combination of empirical and rational evidence-based approaches. The initial empirical derivation of RoSH cell lines from embryos [19] and then E-RoSH lines from ESCs provided the initial wherewithal to develop rational evidence-based approaches for the isolation of E-RoSH cell lines from ESCs. Although these approaches were highly reproducible in our hands, it relied heavily on descriptive parameters and was therefore not easily transferable to other laboratories. Nevertheless, this empirical protocol generated the critical purified cells for a genome-wide panning for candidate genes encoding surface markers that were differentially expressed in RoSH-like cells versus ESCs, and culminated in the identification of CD9^hi^ SSEA-1^−^ cell population as RoSH-like cell population in differentiating EBs.

The identification of CD9 as a surface marker that mark putative RoSH-like cells in a differentiating ESC culture was unexpected as it was not the highest expressing gene in RoSH or E-RoSH relative to ESCs and is a widely expressed gene[31]–[33]. This is further confounded by the observation that several highly expressed genes e.g. Pdgfra and Tie-2 did not produce detectable levels of protein product. The combination of positive selection for high CD9 expression with negative selection against mouse ESC-specific marker, SSEA-1 proved to be highly specific for RoSH-like cells in 5 day old EB-derived cell population as evidenced by the identical gene expression profile and endothelial potential of a CD9^hi^, SSEA1^−^ cell isolate. We observed that CD9^hi^, SSEA1^−^ cells were most abundant in cultures derived from ∼5 days old EBs (data not shown) suggesting that these cells were present transiently during spontaneous differentiation of ESCs,. While differentially expressed CD9 was a good selectable marker for RoSH-like cells in a 5 day old EB-derived cell population, its wide tissue expression in many embryonic tissues[31]–[33] precludes its use as a marker to track putative RoSH-like cells in the developing embryos that have much higher cellular diversity.

CD9 is one of 32 members in the tetraspanin family. Tetraspanins play important roles in numerous various signalling events that affect proliferation, apoptosis, cell morphology, motility and tumour progression[34], [35]. CD9 is expressed in many tissues including hematopioeitic cells e.g B cells[Bibr pone.0000006-Zola1] 31,36, platelets and endothelial cells[37]; early implantation embryos including decidual cells and in the endometrium[38]. It is also expressed on mouse ESCs [28], [32], [39] and we demonstrated here that they were expressed at 10^1–2^ fold lower than in RoSH-like cells. This suggests that the ubiquitously expressed CD9 may be expressed at different levels in a tissue-specific manner. The isolation of a lineage-restricted ESC-derived stem cell line with endothelial-like potential further suggests that CD9 which is associated with many aspects of endothelial functions e.g. leukocyte adhesion and transendothelial migration[40], [41] is expressed at high levels in endothelial progenitors and differentiated progeny. The 89% identity of human and mouse CD9 protein sequences (NCBI HomoloGene database) also suggests that RoSH-like cell lines from human ESCs could be isolated by positive selection for CD9 in conjunction with negative selection for a human ESC-specific surface e.g. SSEA-4.

The derivation of RoSH and E-RoSH cell lines from mouse embryo cultures and ESC-derived EB cultures respectively suggest that differentiation of mouse embryos and ESCs *in vitro* generate RoSH cells as one of the transitional lineage restricted cell types. However, it remains to be determined if RoSH-like cell types are generated during the normal course of embryonic development. Attempts to determine if RoSH cells can participate in embryonic development by injecting RoSH or E-RoSH cells into blastocysts had not generated any chimeric mice (data not shown). More definitive and tissue-specific markers than CD9 will be essential in assessing if RoSH-like cells exist or are formed during normal embryonic development. In addition, the upregulated expression of genes associated with early endodermal and mesodermal differentiation (fig 2g,h) suggests that beside endothelial differentiation, E-RoSH cells may have the potential to differentiate into endodermal and other mesodermal cell types. However, to date we have not been able to generate cell E-RoSH-derived cell types that resembled mature endodermal or other mesoodermal cell types.

In summary, we demonstrate here a methodology to circumvent an impasse in isolating lineage-restricted stem cell lines from ESCs where the isolation of lineage-restricted stem cells is hindered by the lack of defining selectable markers which in turn, cannot be identified for a lack of highly purified lineage-restricted stem cells. The impasse was gradually resolved by first using an empirical approach that relies on the stochastic differentiation of mouse embryos or ESCs to establish lineage-restricted cell lines with endothelial-like potential. These self-renewing cell lines make possible a systematic genome-wide panning for specific selectable markers that distinguish these cells from the parental ESCs. This approach may be applied to the challenging task of isolating and propagating increasing lineage restricted stem cells that generate therapeutically useful cell types such as insulin producing cells.

## Supporting Information

Data S1 Microarray analysis of gene expression in RoSH-like cells vs ESCs. Total RNA (2 µg) from each sample of E-RoSH2.1 p6 and p10, E-RoSH3.2 p2 and p7, RoSH2 p9 and p13, E-RoSHL1 p5 and p6, and E14 mESCs p15 and p16 cultures were converted to biotinylated cRNA using the Illumina RNA Amplification Kit (Ambion, Inc., Austin, TX) according to the manufacturer's instructions. Samples were purified using the RNeasy kit (Qiagen, Valencia, CA). Hybridization to the Sentrix Mouse Refseq-8 Expression BeadChip (Illumina, Inc., San Diego, CA), washing and scanning were performed according to the Illumina BeadStation 500x manual. The data were extracted, normalized and analyzed using Illumina BeadStudio provided by the manufacturer.
(4.12 MB XLS)
